# Tryptophan hydroxylase (TRH) loss of function mutations in *Daphnia* deregulated growth, energetic, serotoninergic and arachidonic acid metabolic signalling pathways

**DOI:** 10.1038/s41598-019-39987-5

**Published:** 2019-03-06

**Authors:** Bruno Campos, Claudia Rivetti, Roma Tauler, Benjamin Piña, Carlos Barata

**Affiliations:** Department of Environmental Chemistry, Institute of Environmental Assessment and Water Research (IDAEA, CSIC), Jordi Girona 18, 08017 Barcelona, Spain

## Abstract

Serotonin has a pivotal function regulating development, growth, reproduction and behavior in animals. In this paper, we studied the deregulatory effects of the deprivation of serotonin in *Daphnia magna* TRH CRISPR-Cas9 mutants. Bi-allelic *in-del* THR mutants and, to a lesser extent, mono-allelic ones grew less, reproduced later, and produced smaller clutches than wild type clones. Transcriptomic and functional gene analyses showed a down-regulation of growth/molting and energy metabolism signaling pathways in TRH mutants, while revealing marked differences between mono- and bi-allelic clones. Bi-allelic mutants, lacking serotonin, presented the serotonergic synapse and arachidonic acid metabolic pathways down-regulated while the tryptophan to kynurenine was upregulated, thus indicating a cross-talk between the serotonergic and arachidonic acid metabolic pathways. Finally, the effects on the insulin growth factor-mediated signaling pathway were marginal. These changes in functional and metabolic pathways are consistent with previously reported effects in *D*. *magna* exposed to pharmaceuticals that inhibited arachidonic metabolism or enhanced the levels of serotonin.

## Introduction

Serotonin plays an important role regulating development, growth, reproduction and behavior in most organisms. Serotonergic neurons promote the production of insect ecdysteroid and juvenile hormones, which control oogenesis and vitellogenesis in arthropods^1–3^. Most studies on the effects of serotonin in crustaceans have been performed using pharmaceutical challenges^4^. In decapod species, serotonergic drugs have been shown to increase ovarian maturation^5^, enhance the crustacean hyperglycaemic hormone that regulates energy metabolism^6^, and modulate aggressive and anxiety-like behaviour^7,8^. In the non-decapod crustacean ecotoxicological and genetic model species, *Daphnia magna*, drugs that enhanced the accumulation of serotonin in the synaptic cleft (such as selective serotonin reuptake inhibitor, SSRIs) increased the immunoreactivity of serotonin in the brain at limiting food environments to higher levels, similar to those observed under non-limiting food environments and, concomitantly, increased fecundity^9^. Physiological, behavioral and transcriptomic studies found that SSRIs enhanced aerobic metabolism in *D*. *magna*, upregulating the Krebs cycle and sugar metabolism while decreasing negative phototaxis^10,11^. In natural conditions, it is an advantage to produce small clutches of larger offspring under food scarcity, since larger offspring perform better under these conditions^12^. Being less tolerant to low oxygen concentrations or being less apprehensive to light is also maladaptive, as *Daphnia* usually migrates during the day to deeper waters in order to avoid fish predation, and often these waters present low oxygen levels in real field conditions^13^. Therefore, previous findings indicated that drugs enhancing serotonin activity at limiting food conditions, originated maladaptive responses in *Daphnia* individuals.

Nonetheless, pharmacological challenges often suffer from additional undesired side effects on other neurotransmitters or/and processes and hence are more difficult to interpret. Thus, the use of directed mutagenesis, when available is a more suitable approach to selectively unravel the functions of serotonin. Recently, we used the CRISPR-Cas9 DNA editing technology to obtain knockout (KO) mutants for the *tryptophan hydroxylase gene* (*DapmaTRH*), which encodes the rate limiting enzyme in the serotonin synthesis pathway. We produced seven TRH mutants in *D*. *magna*^14^. Mono-allelic TRH mutants showed normal levels of serotonin in the brain, whereas  bi-allelic TRH KO clones had no detectable levels of serotonin. Reproduction, growth and phototactic behavior of TRH KO clonal lineages showed the opposed phenotype of those exposed to SSRIs: KO individuals grew less, reproduced later with smaller clutchs and were more responsive to light than wild type clones^14^.

Studies in both *Drosophila* and in the worm *C*. *elegans* indicate a link between serotonin and insulin signalling pathways. Mutations on the *C*. *elegans* insulin receptor gene and on the TRH gene for serotonin synthesis are known to increase reproductive longevity due to the activation of a common insulin and serotonin transcription factor^15,16^. Similarly, reverse genetics experiments showed that a nucleostemin family GTPase acts in serotonergic neurons to regulate insulin signaling and growth in *Drosophila*^17^. The genome of *D*. *pulex*, a close relative of *D*. *magna*, presents at least four insulin/IGF-like receptors and putative insulin related neuropeptides^18,19^. The genome of *D*. *pulex* also encodes other key insulin-related downstream elements, including several lipases, kinases, docking proteins (i.e. the insulin receptor substrate) and transcription factors (i.e. the forkhead transcription factor FOXO)^18^. Nevertheless, previous efforts to detect insulin immuno-reactivity in the central and peripheral neurological system of *D*. *magna* have been so far unsuccessful (Dircksen & Campos, unpublished data).

Another proposed physiological role of serotonin is the regulation of the arachidonic acid and eicosanoid metabolism, which play vital roles in *Daphnia* reproduction and growth^20–24^. Once released from presynaptic axonal terminals, serotonin binds to several family receptors. One of them, the 5-HT2 (G protein-coupled) receptor family, modulates phospholipase A2, which is responsible to release arachidonic acid from phospholipids in humans and other vertebrates. Genome sequence analyses as well as data from transcriptomic and metabolomics studies in *D*. *pulex* and *D*. *magna* suggest the presence of the genes encoding for phospholipase A2 and prostaglandin metabolic enzymes in daphniids^22^.

The main objective of this work is to study the molecular mechanisms by which serotonin regulates growth, reproduction, and behavior in *D*. *magna*.

This was accomplished comparing transcriptional profiles of serotonin-deficient *D*. *magna* adult individuals with the wild type, profiting of three previously described TRH CRISPR-Cas9 mutant clones. Two of these mutants were encompassing bi-allelic *in-del* mutations (TRHA−/− and TRHB−/−, hereafter named TA−, TB−, respectively), whereas the third presents a mono-allelic *in-del* TRHA −/+ clone (hereafter named as T+) and was used as a positive control^14^. TA− and TB− clones present no functional copies of the tryptophan hydroxylase gene (*DapmaTRH*), therefore lacking serotonin; whereas T+ maintains an intact TRH allele and shows normal levels of serotonin^14^. We aimed to identify gene pathways related to the observed physiological and phenotypic differences between these clones and to search for the link between serotonergic, arachidonic/prostaglandin and insulin signaling pathways. We performed a non-targeted transcriptomic approach to search for unknown altered gene signaling pathways, using a recently developed and validated new custom-made microarray that includes probes from the full transcriptome of *D*. *magna*^25^. Differentially transcribed genes were related to effects observed on growth and reproduction.

## Results

### Life-history responses

The age and body length at first reproduction and total offspring production were significantly (P < 0.05) affected in all *in-del* mutated TRH clones relative to the wild type one, although effects were less pronounced in clone T+ (Table 1, stats in Supplementary Table S1).

**Table 1:** TRH mutated clones grew less and bi-allelic TRH ones reproduced less and latter.

Clone	Age	Body length	Fecundity
Wild	8.5 ± 0	2639.6 ± 21.2	50.6 ± 0.6
T+	8.7 ± 0.1	2506.5 ± 39.3*	46.3 ± 1.5
TA−	9.3 ± 0.1*	2440.5 ± 44.5*	40.7 ± 2.6*
TB−	9.3 ± 0.1*	2437.7 ± 39.1*	40.3 ± 1.6*

Age and body length at first reproduction and total offspring production (Mean ± SE, N = 9–10) of the four studied clones. *Significant (P < 0.05) differences from the wild type clone following ANOVA and Dunnett’s or equivalent non parametric tests.

### Transcriptomic analysis

**Figure 1:**
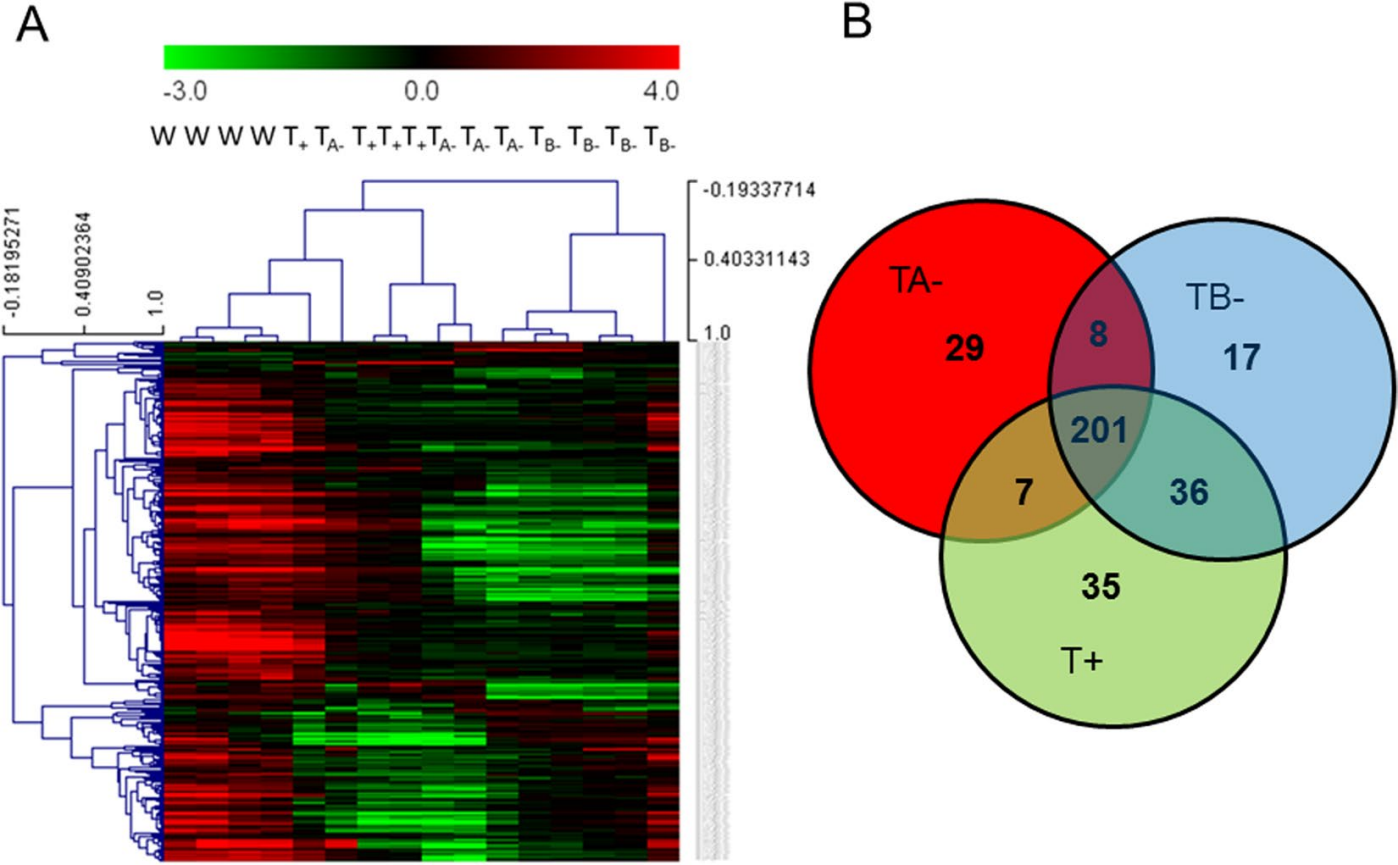
Analysis of microarray results. (**A**) Heat map and Pearson hierarchical clustering of the 147 transcripts identified as underrepresented in CRISP-Cas9 TRH clonal mutants relative to the wild type clone across the four individual-replicates per clone. (**B**) Venn diagram showing the overlapping of transcriptomic effects between individuals of the three mutant clones. T+, TA−,TB− refer to one mono-allelic and to two bi-allelic TRH mutant clones, respectively.

Normalized fluorescence values of 333 probes belonging to 252 unique annotated genes were significantly different (P < 0.05) in mutated clones relative to the wild type. Most of these probes (313 probes, belonging to 236 annotated genes) appeared underrepresented in CRISPR/Cas9-mutated clones relative to the wild type by a ≥1.5 Fold Change. In contrast, only T+ and TA− clones showed a small group of over-expressed genes (20 probes, representing 16 unique genes) by more tan ≥1.5 fold (down and up regulated probes in each studied clones are depicted in Supplementary File S1). Hierarchical clustering of the fluorescence values of each of the four individual-replicates per clone for the 333 down-regulated probes showed a clear separation of wild type individuals from mutated ones, and that TB− individuals were also forming a distinct cluster, together with some probes from TA− individuals (Fig. 1A). Figure 1A also shows a consistent response pattern of most of the four individual-replicates per clone grouping together. A Venn diagram shows that most down-regulated probes were common for the three CRISPR/Cas9 mutants (201 de-regulated probes, 147 annotated genes, Fig. 1B), and that few of these probes (35, 29 and 17 for clones T+, TA− and TB−, respectively) were clone-specific.

**Figure 2:**
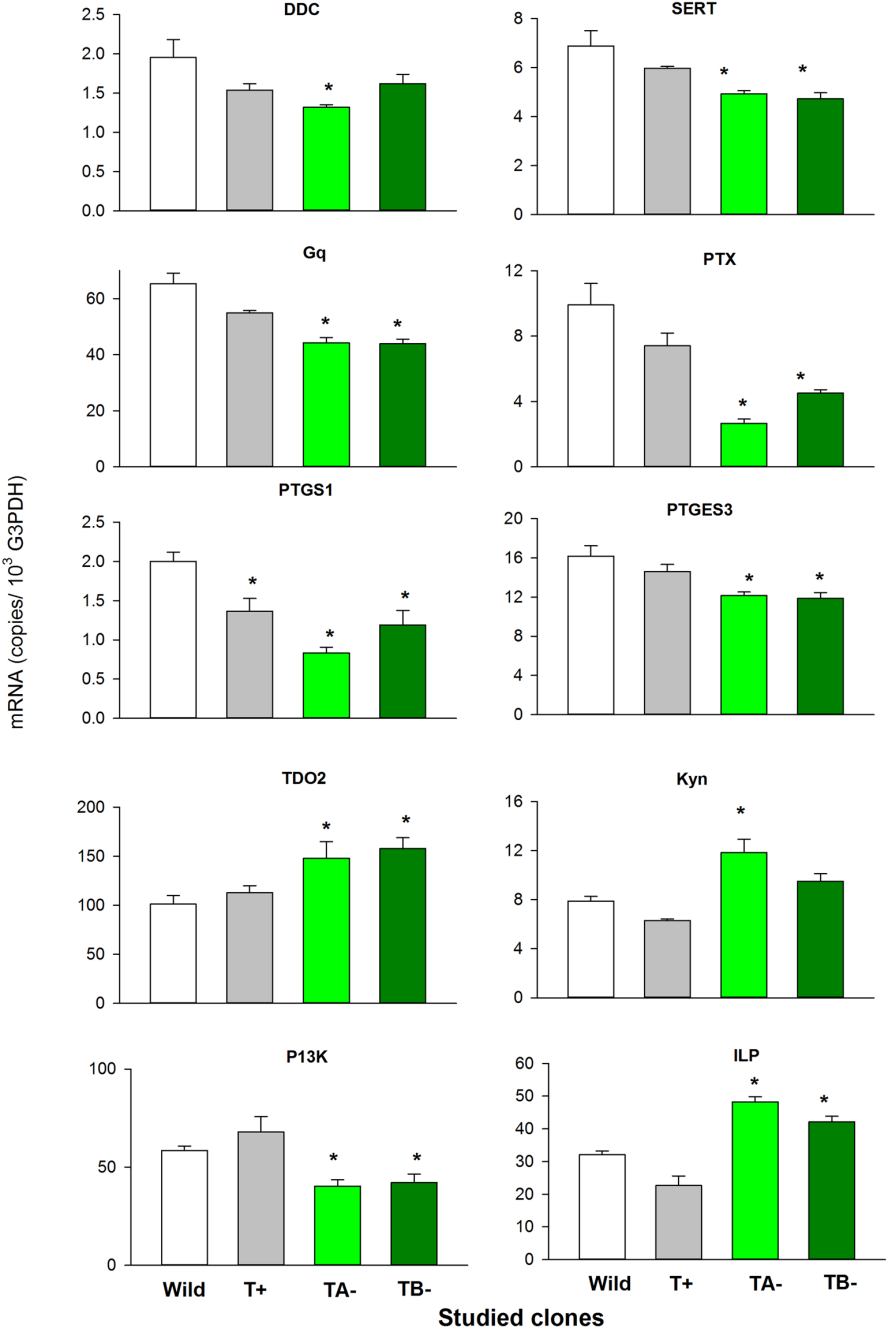
Transcript abundance of selected genes, as determined by qPCR. The bars represent mRNA abundances in copies per 1000 copies of G3PDH mRNA (Mean ± SE, N = 4). DDC is involved in serotonin synthesis, SERT codifies a transporter implicated in the serotonin re-uptake mechanism, Gq, PTX, PTGS1, and PTGES3 are related to the arachidonic/prostaglandin synthesis pathway, TDO2 and Kyn codify enzymes for the synthesis of kynurenine from tryptophan, and PI3K and IL3 are related to the insulin growth factor signalling pathway. *Significant p < 0.05 differences relative to the wild type following ANOVA and Dunnet’ s test. ANOVA results are depicted in Supplementary Table S1. White, grey, light and dark green triangles correspond, respectively, to control, T+, TA− and T− clones, respectively.

**Figure 3:**
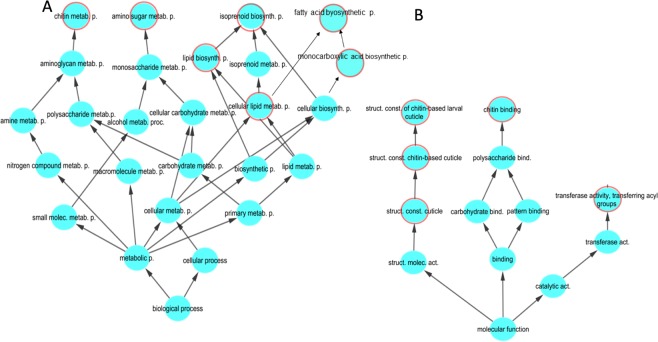
Gene ontology network analysis. Significantly enriched terms (P < 0.05) are labelled with a red circle. The analysis included biological processes and molecular function GO terms for all genes down-regulated in all three CRISP-Cas9 TRH mutated clones (for further details see Table 2). The data indicates that the three mutated clones had down-regulated molecular/biological processes related to growth (cuticle, chitin) and energetic metabolism.

Gene ontology analysis indicated that the 147 transcripts showing under-representation in all three CRISPR-Cas9 mutants were related to growth (cuticle, chitin), energetic metabolism (amino sugar, fatty acid, lipid metabolism), and secondary metabolism (transferase activity) GO terms (Fig. 2, Table 2). When looking to the commonly de-regulated genes across CRISPR-clone-specific we identified 15 genes belonging to different specific regulatory signals or metabolic pathways related with serotonin synthesis and re-uptake, arachidonic/prostaglandin metabolism, insulin like growth factor signaling and the tryptophan catabolic process to kynurenine. Supplementary Figure S1 shows the behavior of the 15 transcripts identified in the microarray and annotated in one of the above mentioned functional categories. qPCR analyses performed on these 15 transcripts across the four studied individual/replicates per clone confirmed that only in 10 of these genes the observed microarray normalized fluorescence values correlated significantly (P < 0.05) with the mRNA abundance (Supplementary Fig. S2). Figure 3 shows the qPCR mRNA levels of the genes that varied significantly (P < 0.05) across clones following ANOVA analyses (ANOVA results are depicted in Supplementary Table S1). qPCR mRNA levels of genes belonging to the serotonin synthesis and re-uptake (DDC, SERT), arachidonic/prostaglandin metabolism (Gq, PTGS1, PTX, PTGES3) were down-regulated in clones TA− and TB−, those of genes from the tryptophan/Kynurenine metabolism (TDO2, Kyn) were up-regulated, whereas insulin-like growth factor signaling genes (PI3-kp85/p60, ILP) were up or down regulated. As all these effects were observed only in clones having mutations in both alleles (TA−, TB−), leading us to propose that they reflect the specific effects of serotonin deprivation.

**Table 2:** The three mutated clones had down-regulated molecular/biological processes related to growth (cuticle, chitin) and energetic metabolism (amino sugar, fatty acid, lipid metabolism) and secondary metabolism (transferase activity).

		T+ D		TA− D		TA−U		TB− D		Common D		T+ Unique	
GO ACC	GO Term	Count	%	Count	%	Count	%	Count	%	Count	%	Count	%
**Molecular function**													
GO:0042302	Cuticle constituent	11	9.7	8	8.8			10	9.5	8	9.8		
GO:0008061	Chitin binding	9	7.9	6	6.6			6	5.7	5	6.1	4	14.8
GO:0005214	Chitin constituent	10	8.8	8	8.8			10	9.5	8	9.8		
GO:0008010	Structural constituent of chitin-based larval cuticle	10	8.8	8	8.8			10	9.5	8	9.8		
GO:0016746	Transferase activity(acyl)	8	7.1	5	5.5			6	5.7	7	8.6	2	7.4
**Biological Process**													
GO:0006040	Amino sugar metabolism	8	7.1	8	8.8			7	6.7	7	8.6		
GO:0006022	Aminoglycan metabolism	7	6.2	5	5.5			5	4.8	5	6.1	2	7.4
GO:1901071	Glucosamine metabolism	11	9.7	8	8.8			7	6.7	7	8.6	3	11.1
GO:0008610	Lipid biosynthesis	11	9.7	9	9.9			9	8.6	7	8.6	2	7.4
GO:0006633	Fatty acid biosynthesis	7	6.2	5	5.5			6	5.7	4	4.9	2	7.4
GO:0008299	Isoprenoid biosynthesis	2	1.8	3	3.3			3	2.9	3	3.7		
GO:0072330	Monocarboxylic acid biosynthesis	7	6.2	4	4.4			6	5.7	4	4.9	2	7.4
GO:0006030	Chitin metabolic process	6	5.3	5	5.5			4	3.8	4	4.9	2	7.4
GO:0042335	Cuticle development	12	10.6	7				11	10.5	7	8.6		
GO:0044255	Cellular lipid metabolism	10	8.8	11	12.1			10	9.5	10	12.3		
GO:0019441	tryptophan catabolic process to kynurenine					2	15.4						

Significant (P < 0.05, after false discovery rate correction) of gene enriched signalling pathways of the deregulated probes across the gene clusters identified in the Venn diagram. Only the number of unique probes and %coverage are reported. Full set of genes are depicted in Supplementary File S2.

**Figure 4:**
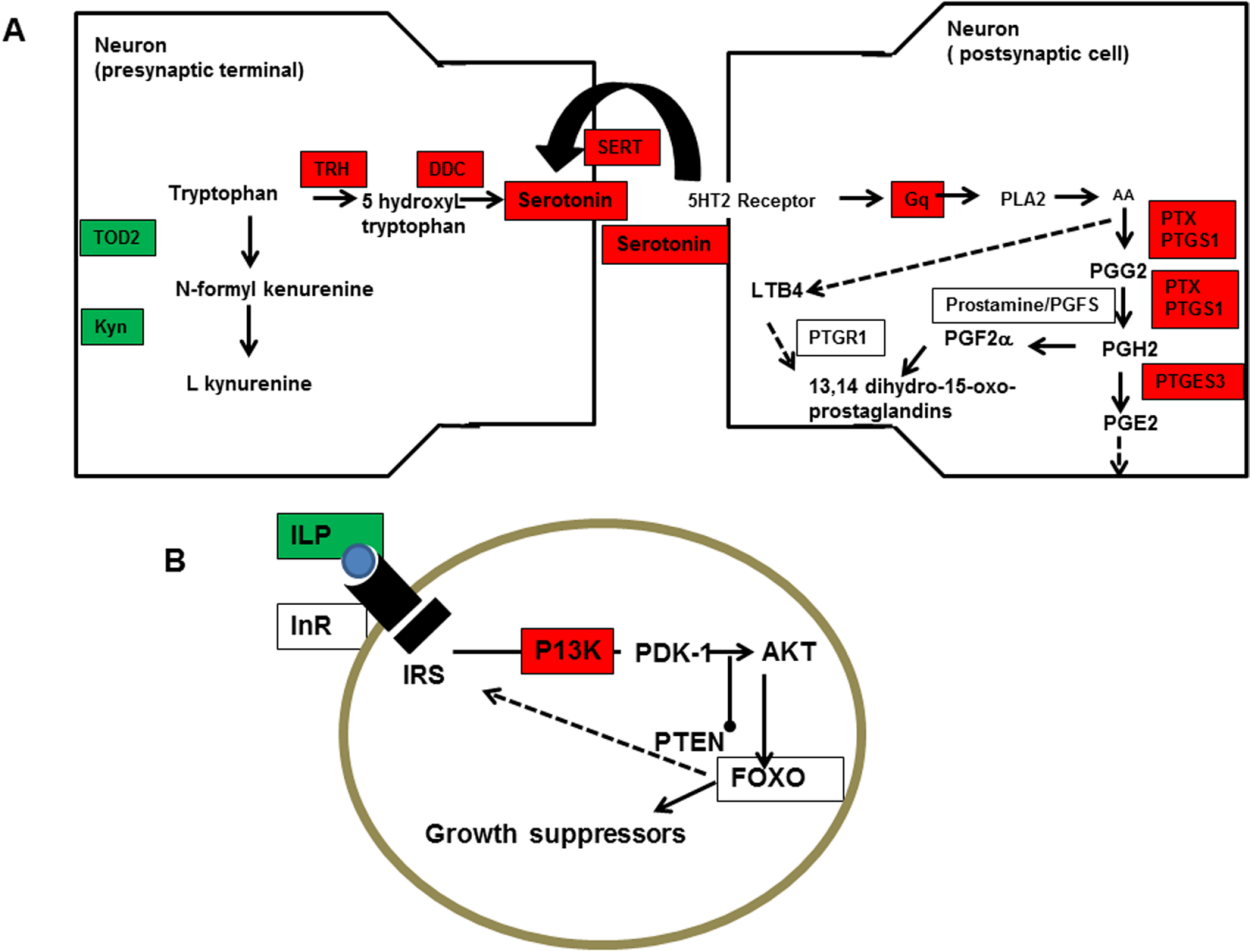
Schematic representation of the metabolic/physiological intercorrelation of identified de-regulated genes in Bi-allelic mutated TRH clones. The scheme represents both the pre- (left) and the post-synaptic (right) neuron of a putative serotoninergic synapsis (**A**) and the insulin signalling pathways (**B**). Outlined pathways are adapted from Heckman *et al*.^21^, Boucher *et al*.^18^, Edgar^36^ and Tootle and Spradling^30^ ones. De-regulated Genes/enzymes/neurotransmiters are shown in red (down) or green (up) boxes, those identified as de-regulated in the microarray but not confirmed by qPCR are depicted in white boxes.

Figure 4 shows the functional and physiological correlation between the affected signaling and metabolic pathways. Note that three of them (serotonin synthesis and re-uptake, arachidonic/prostaglandin metabolism, and kynurenine synthesis) are intimately related to the serotoninergic synaptic function.

## Discussion

This study aimed to study the signaling pathways affected by serotonin in *D*. *magna* by comparing transcriptional patterns of two distinct CRISPR/Cas9 bi-allelic *in-del* TRH mutants lacking serotonin with other two clones showing normal levels of serotonin, the wild type clone and a CRISPR/Cas9 mono-allelic TRH mutant^14^. The study of gene transcription patterns showed that most de-regulated genes were common for both mono- and bi-allelic mutants, and that the vast majority of them were under-represented in all three mutants, relative to the wild type. The functional analysis of these genes denoted de-regulation of routes related to growth/molt and energetic metabolism. These results are in line with the observed delayed reproduction, reduced body size and fecundity observed in mutated clones, despite the fact that these effects in bi-allelic clones were stronger than in mono-allelic ones. Conversely, previous analyses of transcriptomic changes associated to exposure to drugs/chemicals that enhance, rather than inhibit, reproduction and growth revealed up-regulation of energetic metabolic pathways^10^. Thus, both sets of data show a clear and consistent correspondence between transcriptomic and life-history changes.

The study of genes from metabolic routes closely related with serotonin denoted marked differences between clones deprived of serotonin (TA−, TB−) and those showing normal levels of it (T+, W). For example, the clones lacking functional TRH alleles showed a down-regulation of the chromosomic *DDC* gene, which encodes the enzyme immediately downstream from *TRH* for serotonin synthesis from tryptophan (see Fig. 4). Campos, *et al*.^10^ found that the DDC gene was upregulated by drugs that enhanced serotonin signaling, a response exactly opposite to the one observed here. In addition, serotonin-deprived clones showed a decrease of transcripts encoding *SERT*, the transporter involved in serotonin transport, removing it from the synaptic cleft^26^, a metabolic response consistent with the abolition of the serotonergic function (Fig. 4). Finally, the observed up-regulation of the two genes encoding the enzymes for kynurenine synthesis in TA− and TB− clones (see Fig. 4) can be regarded as a compensatory metabolic mechanism, as the synthesis of kynurenine from tryptophan is considered a mechanism of disposal of any excess of tryptophan in the brain^27^. Therefore, the data is consistent with a model in which the lack of functional TRH enzyme results in an excess of tryptophan in the brain and the activation to metabolic pathways able to compensate it (see Fig. 4).

In addition, the complete deprivation of serotonin in TA− and TB− clones lead to the down-regulation of several genes that can be related to the serotonin response in the post-synaptic neurons. For example, the guanine nucleotide-binding protein *G(q)* subunit alpha, which belongs to the 5-HT2 (Gq-coupled) family receptors, is activated by the 5HT2 serotonin receptor and, in turn, activates the arachidonic acid/prostaglandin pathway^28,29^. Up to six genes in this pathway appeared down-regulated in TA−, TB− clones (four of them confirmed by qPCR), consistent with the abolition of serotoninergic signaling in these mutant clones. Heckmann, *et al*.^22^ first outlined the structure of eicosanoid biosynthesis in *Daphnia* and their modulation with ibuprofen, a known inhibitor of the rate-limiting enzyme for prostaglandin synthesis. In the previous study, PTGS1, also known as COX, showed a close relationship between the inhibition of the prostaglandin pathway and reproduction^20,21^. In *Drosophila* there is also a COX homologue, PTX, which is a cyclooxygenase-like facilitator of follicle maturation^30^. Both genes, PTGS1 and PTX, together with, PTGES3, also involved in the synthesis of at least three different prostaglandins (PGG2, PGH2, PGE2), were down-regulated in serotonin-deprived clones (Fig. 4). Thus, our results provide for the first-time transcriptomic evidence supporting the argument that there is a cross-talk between serotonin and arachidonic acid/prostaglandin pathways in *Daphnia*, and that the down regulation of these pathways may explain observed effects on growth and reproduction in serotonin-deprived clones.

However, prostaglandin signaling in insects has been mainly associated with reproduction, and not with growth^31^. A connection between serotonin and insulin signalling has been described in *C*. *elegans* and *Drosophila*. In *Drosophila*, a nucleostemin family GTPase acts in serotonergic neurons to regulate insulin signaling and control body size^17^. The genome of *D*. *pulex*, a close relative of *D*. *magna*, have four genes for insulin/IGF-like receptors and also genes encoding other key insulin related elements downstream such as several lipases, kinases, docking proteins (i.e. the insulin receptor substrate), transcription factors (i.e. the forkhead transcription factor FOXO) and putative insulin related neuropeptides^18,19^. Our microarray analysis indicated overrepresentation of FOXO, ILP and IR transcripts, and a reduction of PI3-kp85/p60 ones, although these results were only partially confirmed by qPCR (Fig. 4). This means that our transcriptomic data provides only marginal evidence indicating that serotonin affected growth through the de-regulation of the insulin/IGF-like signalling pathway. Thus, more research is needed to unravel the cross-talks between serotonin and insulin signalling pathways in *Daphnia*.

In summary, our results provide additional experimental evidence showing that the CRISPR/Cas9 methodology is a powerful tool to introduce reverse genetics and hence, to study the function of specific gene/enzymes in many species previously considered as inaccessible. In this paper, we used this approach to study the transcriptional changes associated to the knockout of a key gene/enzyme involved in the synthesis of serotonin. Observed transcriptomic changes showed common gene response patterns among the studied mutated clones, which correlated to phenotypic changes such as reduced growth and reproduction. However, several of the observed de-regulated genes were specific of mutants lacking serotonin, which included down-regulation of the serotonin synapsis and arachidonic acid metabolism, and up-regulation of the kynurenine pathway. These results agree with previously reported transcriptomic effects in the arachidonic acid, serotonin and energetic metabolic pathways of *D*. *magna* individuals exposed to ibuprofen or selective serotonin reuptake inhibitory drugs^10,21^.

## Methods

### Experimental animals

An extensively characterized single clone of *D*. *magna* (clone F)^32^, hereafter designated as wild type clone (W), was used as the source for generating the three studied CRISPR/Cas9 -mediated TRH mutant clonal lines: T+(mono-allele *in-del* mutant) and TA− and TB− (bi-allele *in-del* mutants). Further details about the generation of these TRH mutant clonal lines and their respective phenotypes have been previously reported by Rivetti, *et al*.^14^.

Individual or bulk cultures of 10 animals/l for each clone were maintained in ASTM hard synthetic water at 20 °C under a 16 h light: 8 h dark photoperiod and fed daily with the algae *Chorella vulgaris* (5 × 10^5^ cells/ml) following previously described procedures^32^.

### Reproduction

Effects on reproduction were assessed following the established OECD guidelines with minor modifications^32^. Assays were conducted with the selected three TRH mutated clones plus the wild type one (W). Newly born neonates (<24 h old) were reared individually in 100 mL of ASTM hard water at high food ratio conditions (5 × 105 cells/ml of *C*. *vulgaris)* until they release the third brood. Each treatment was replicated 10 times. The test medium was changed every other day. Measured life-history traits included body length, age at first reproduction and total offspring production. Body length measurements were performed following previously described procedures^14^.

### Transcriptomic analyses

At the end of reproduction experiment adult females of each clone were de-brooded as previously described, snap frozen in liquid N_2_ and stored at −80 °C until RNA extraction^10^.

### RNA Extraction

Total RNA from a single adult *D*. *magna* female was isolated using Trizol (Invitrogen, USA), following manufacturer protocols with slight modifications. After RNA isolation, DNAse treatment was performed according to manufacturer protocols, followed by a double phenol-chloroform and another chloroform extraction for further purification. RNA was precipitated using sodium acetate and 100% ethanol, being re-suspended in RNAse free water, and finally quantified and quality checked in a NanoDrop D-1000 Spectrophotometer (NanoDrop Technologies, USA). Samples presenting a ratio 230/260–260/280 between 1.9–2.1 were selected. RNA integrity was checked using Agilent 2100 Bioanalyzer (Agilent Technologies, USA). Only the samples showing RIN values above 9 were used for microarray analysis.

### Microarrays

A 8 × 60 K Agilent array containing the full set of the 41317gene models representing the full transcriptome of *Daphnia magna* was used^33^. This platform was designed from a previous 4 × 180 K one (Agilent 66414 design; GPL22721), which contained four probes per gene model and that was tested across seven life-stages^25^. Most probes (39000) included in the 8 × 60 K new platform belonged to unique genes that scored the maximal fluorescence signal across *D*. *magna* life-stages^25^. The array also included the two best probes having the highest signal for the remaining 2317 genes, which showed a less consistent signaling pattern across life-stages. Further e-array based quality controls were added, resulting in a microarray with 50,000 probes, as well as an extra 3500 negative probes, computer generated. This was then printed on an 8 × 60 K format (Agilent 079797design; GPL23826).

A total of four replicates per clone were used. Each replicated contained the RNA extracted from a single adult individual. One μg of total RNA was used for all hybridizations. cDNA synthesis, cRNA labeling, amplification, and hybridizations were performed following the manufacturer’s kits and protocols (Quick Amp labeling kit; Agilent, Palo Alto, CA). The Agilent one-color Microarray Based Gene Expression Analysis v6.5 was used for microarray hybridizations according to the manufacturer’s recommendations. Microarray images were generated by an Agilent high-resolution C microarray scanner. Data was resolved from microarray images using Agilent Feature Extraction software v10.7. Raw microarray data from this study have been deposited at the Gene Expression Omnibus Web site (www.ncbi.nlm.nih.gov/geo/) with accession number GSE101858.

### Gene expression analysis

Microarray data were analyzed using Gene Spring GX v13.0 software (Agilent, USA). Fluorescence data was normalized using quantile normalization and baseline transformation to the median of all samples.

The quantile 95 of the added 3500 negative probes was calculated and this value was assumed as being the fluorescence background noise value of each sample.

Differentially transcribed probes (DEP) of four *D*. *magna* adult individuals from the three CRISPR/Cas9 clones (T+, TA−, TB−) relative to those of the wild type clone (W) were identified by comparing normalized fluorescent levels using one way ANOVA test (p < 0.05) followed by a Dunett’s test plus Benjamini-Hochberg false discovery rates correction and using a 1.5 fold change cut-off. The latter term was calculated as the quotient between the antilog2 of the replicated normalized fluorescence of CRISPR/Cas9 clones versus mean normalized fluorescence of the four replicates of the wild type clone. Only those probes from CRISPR/Cas9 clones whose absolute normalized fluorescence changed significantly ≥1.5 fold relative to the wild type clone were considered up or down regulated. Finally, differentially transcribed genes were those associated to DEP. Sample Clustering of DEP across the studied clones was analysed using the Multi-Experiment viewer MeV4 software by hierarchical clustering using Pearson correlation algorithm^34^. Finally a Venn diagram was used to represent common and unique DEP across the studied genes.

Differentially annotated transcribed genes were used to assess enriched functional gene ontology and metabolic signalling pathways using the GeneSpring Enrichment tool and the Kyoto Encyclopaedia of Genes and Genomes (KEGG, http://www.genome.jp/kegg/kegg2.html) database. The latter was restricted to the specific signaling pathways related with tryptophan/serotonergic, arachidonic acid metabolism, and insulin like growth.

### Validation of microarray results by qPCR

Microarray results were validated with real-time quantitative polymerase chain reaction (qPCR), which was performed using the same samples (four individual/replicates per clone) used in the array. Validation was performed comparing microarray normalized fluoresce values with normalized qPCR mRNA abundance, both log e transformed. We selected 15 differentially expressed genes belonging to the tryptophan/serotonergic synapse (tryptophan 2,3-dioxygenase A, TDO2; kynurenine formamidase, Kyn; dopamine decarboxylase, DDC; serotonin transporter, SERT; G protein subunit alpha q, Gq;, arachidonic acid (prostaglandin E2 receptor EP4 subtype, PTGER4; prostaglandin-endoperoxide synthase 1, PTGS1 or COX1; cyclooxygenase-like facilitator of follicle, PXT; Prostamide/prostaglandin F synthase, PGFS; Prostaglandin reductase, PTGR1; Prostaglandin E synthase 3,PTGE3) and insulin growth factor signaling pathways (Insulin like peptide, ILP; Insulin receptor, IR; phosphatidylinositol 3-kinase, PI3-kp85/p60 and Forkhead transcriptional factor FOXO^10,18,22,27,30,35^). The G3PDH gene (glyceraldehyde 3-phosphate dehydrogenase) was used as an internal control (house-keeping gene). Primers for each one of these genes were designed with Primer Express® Software v3.0.1(Thermofisher, USA) and are provided in Table S1. qPCR was performed according to manufacturer’s protocols.

## Supplementary information

Supplementary information,

File S1,

File S2
