# Supplementary material for: Tryptophan hydroxylase (TRH) loss of function mutations in Daphnia deregulated growth, energetic, serotoninergic and arachidonic acid metabolic signalling pathways
Source: Sci Rep. 2019 Mar 6;9:3693. doi: 10.1038/s41598-019-39987-5 (PMC6403212; doi:10.1038/s41598-019-39987-5)
Supplement: Supplementary file 1 — Supplementary information, [file 41598_2019_39987_MOESM1_ESM.docx]

**SUPPLEMENTARY INFORMATION**

Tryptophan hydroxylase (TRH) loss of function mutations in *Daphnia* deregulated growth, energetic, serotoninergic and arachidonic acid metabolic signalling pathways

Bruno Campos, Claudia Rivetti, Romà Tauler, Benjamin Piña, Carlos Barata

**Supplementary Tables**

**Supplementary Table S1**. **Statistical results**.

|  | df | X^2^ or F | p |
| --- | --- | --- | --- |
| Life history traits |  |  |  |
| Age* | 3 | 25.6 | <0.001 |
| Body length** | 3,36 | 6.5 | 0.001 |
| Offspring production** | 3,35 | 8.1 | <0.001 |
| Genes |  |  |  |
| DDC** | 3,12 | 11.1 | 0.001 |
| SERT** | 3,12 | 7.8 | 0.004 |
| Gq** | 3,12 | 8.4 | 0.003 |
| PTX** | 3,12 | 14.2 | <0.001 |
| PTGS1** | 3,12 | 8.9 | 0.002 |
| PTGES3** | 3,12 | 8.3 | 0.003 |
| TDO2** | 3,12 | 6.8 | 0.006 |
| Kyn** | 3,12 | 26.8 | <0.001 |
| P13K** | 3,12 | 7.2 | 0.005 |
| ILP** | 3,12 | 20.5 | <0.001 |

Kruskal-Wallis* and one-way ANOVA** results comparing the performance of the four studied clones. df, χ2, F, p are degrees of freedom, Chi-square, Fisher’s coefficient and probability level, respectively.

| Table S2. Primer pairs designed from existing sequences used for amplification of selected *Daphnia magna* partial gene sequences. | | | | | |  |  |
| --- | --- | --- | --- | --- | --- | --- | --- |
| Name | code | access number | Forward | Reverse | | | Amplicon (bp) |
| Guanine nucleotide-binding protein G(q) subunit alpha | Gq | KZS13216 | GGAACTAGTCCGGTCCGTTG | CTCCACAAGTCCCGTATGGC | | | 81 |
| Chorion peroxidase | PXT | KZS13261 | CCCGTCGTGCAGAACTGAC | AAGCCGGAAAGATCCAGGAA | | | 81 |
| Sodium-dependent serotonin transporter | SERT | KZS12304 | TTCAGTTTTGCTTTAGATTCGGC | CAATAAATATGCGGCTCCACC | | | 81 |
| Kynurenine formamidase | Kyn | KZS09367 | GGAAAATGACCCTCCCGAGT | TGAACATCCGTGAATCCAGC | | | 81 |
| Tryptophan 2,3-dioxygenase A | TDO2 | KZS06758 | CGATTCTGCGAAGAGGGTTG | GCCGCGTAAACACATTCCAC | | | 81 |
| Prostaglandin G/H synthase | PTGS1 | KZS18300 | AGTGCGGCCATATTGGATTC | GATGGACGTCTTGTCGGGAC | | | 81 |
| Dopamine Decarboxylase | DDC | KZS20105 | AATGATTCCTGAAGCCGCC | CCAGGCATAATGACGCGTT | | | 81 |
| Prostaglandin E 3 Synthase | PTGES3 | KZS11998 | GGCTACCAGCAACTCAACGAC | CTGAAGGTCTTGTTGGGCATC | | | 81 |
| Prostaglandin E2 receptor EP4 subtype | PE2REP4 | KZS02991 | CACTTGAATCCATCCGTGCA | CATTATCAGGGCGGCAATGT | | | 81 |
| Prostaglandin E synthase 3 (Cytosolic) | PEsyn3Cyt | A0A0P6AAV6 | TTAATAGATGGCGCGATGAGG | GCATCATCTCGTCGAGGTCC | | | 81 |
| Prostamide/prostaglandin F synthase | PFsyn | A0A0P5H856 | GTTGAGTCGAATTGCTCGCA | TGGTAACCATCTCCGCGTAGA | | | 81 |
| Insulin growth factor receptor | InR | KZS17898 | CCGTCAAGATTGGCGACTTC | ACCAACCTTGTGCGTCTCCT | | | 81 |
| Phosphatidylinositol 3-kinase, PI3-Kp85/p60 | PI3-Kp85/p60 | KZS11075 | GAATGGCCGGCGTAGTAGAC | AGAGAGAGGGTTCGGCTTCAC | | | 81 |
| Insulin/IGF/relaxin peptide 1-like protein | ILP | KZS08602 | GAAAGCTATTGCGCAGTCATCA | CGTCATCAAGGCAGCGCT | | | 81 |
| Forkhead transcription factor FOXO | FOXO | KZS16202 | CGGTGTCGTGTCTGGCTTG | ACGTTGCAATCTTCAAATCCG | | | 81 |
| HK glyceraldehyde-3-phosphate dehydrogenase | G3PDH | AJ292555 | GACCATTACGCTGCTGAATACG | CCTTTGCTGACGCCGATAGG | | | 100 |

**Supplementary Figures**


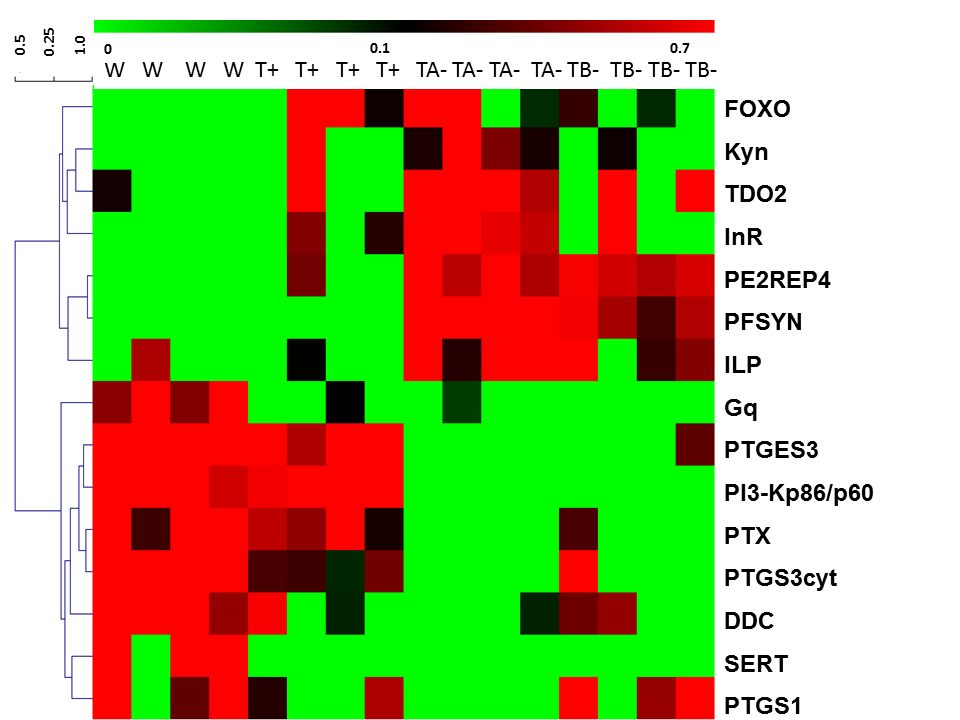


**Fig S1. Hierarchical clustering of fluorescence values (log 2) of deregulated probes encoding for genes belonging to the tryptophan/serotoninergic, arachidonic acid metabolism and insulin growth factor signalling pathways**. Gene abbreviations are explained in Table S1





**Fig S2. Bi-plots of qPCR mRNA abundance relative to G3PDH vs microarray fluorescence values (all log2) (Mean SE, N=4) of the genes depicted in Fig S1.** Only genes with significant Person correlation values are depicted. White, grey, light and dark green triangles correspond, respectively, to control, T+, TA- and TB- clones. *0.05<p<0.01; **p<0.01
